# Structural and Genomic Bases of Branching Traits in Spur-Type Apple: Insights from Morphology and Whole-Genome Resequencing

**DOI:** 10.3390/genes17010096

**Published:** 2026-01-18

**Authors:** Han Wang, Dongmei Chen, Guodong Zhao, Da Zhang, Xin Liu, Bowei Zhu, Linguang Jia, Tongsheng Zhao, Chaohong Zhang, Xinsheng Zhang

**Affiliations:** Changli Institute of Pomology, Hebei Academy of Agriculture and Forestry Sciences, Qinhuangdao 066600, China; 15830298656@163.com (H.W.); chendm2009@126.com (D.C.); guodong19823@163.com (G.Z.); d.zhang@nwafu.edu.cn (D.Z.); 18830180711@163.com (X.L.); 18331561549@163.com (B.Z.); dsjialinguang2020@163.com (L.J.); tshzh71@163.com (T.Z.); zhangxs72@126.com (X.Z.)

**Keywords:** *Malus domestica* Borkh., branching, spur-type, whole-genome resequencing

## Abstract

Background: Plant architecture, particularly branching patterns, plays a crucial role in plant growth, photosynthetic performance, and yield. Spur-type apple, characterized by compact growth, early fruiting, high productivity, and manageable canopy structure, represent valuable germplasm for establishing dwarf and high-density apple orchards. While hybrid breeding of spur-type varieties offers significant potential for genetic advancement, severe segregation of traits in hybrid progeny and the difficulty of combining multiple favorable traits still significantly limit breeding efficiency. Moreover, the genetic basis and molecular mechanisms of the spur-type trait remain poorly understood at the genomic level, hindering the development of precise molecular breeding approaches. Methods: To address this, we used the spur-type line ‘0301-13-14’ and the non-spur-type line ‘0301-50-32’ from hybrid progenies of the spur-type cultivars ‘Miyazaki Spur Fuji’ and ‘Starkrimson’ to elucidate the regulatory mechanisms underlying apple branch formation and spur-type trait development by characterizing their branching traits, performing whole-genome resequencing analysis, and identifying candidate genes using bioinformatics analyses. Results: Anatomical observations revealed that the spur-type line ‘0301-13-14’ possessed smaller cells with a more compact spatial arrangement compared to the non-spur-type line ‘0301-50-32’. Whole-genome resequencing generated 5,003,968 high-quality single-nucleotide polymorphisms (SNPs) and 577,886 high-quality insertions/deletions (InDels). We further identified 29,157 candidate genes harboring predicted deleterious mutations (classified as high or moderate impact). Gene Ontology (GO) enrichment analysis indicated that genes associated with the spur-type trait were mainly enriched in molecular function and biological process categories. Specifically, variant genes related to molecular function were enriched in transferase and catalytic activities, while those in biological process were mainly involved in phosphorylation and phosphorus metabolism. Kyoto Encyclopedia of Genes and Genomes (KEGG) pathway enrichment analysis showed that candidate genes were significantly enriched in environmental information processing and metabolic pathways. Conclusions: These results will provide a genomic foundation for identifying genes controlling spur-type branching traits and facilitate the genetic improvement of spur-type apple.

## 1. Introduction

Branching is crucial for shaping the aboveground structure of plants. It directly influences canopy size and the light-use efficiency of leaves; determines tree yield and fruit quality; and affects the efficacy of cultivation systems and orchard management efficiency [1,2]. The development of lateral branches is a key factor shaping overall plant architecture and affects both growth patterns and resource allocation. In higher plants, shoot branching depends on the formation of axillary meristems and the subsequent growth of axillary buds [3,4]. Axillary bud growth involves bud germination (breaking dormancy) followed by bud elongation. The former determines the number of branches, whereas the latter directly affects branch length. This process is regulated by multiple factors, including environmental conditions [5], plant hormones [6], and genetic determinants [7].

Light functions both as an energy source and as a signal regulating branching. Plants can perceive different light parameters and adjust bud outgrowth and branch development accordingly [8]. In general, increased light intensity promotes lateral bud growth and branch elongation in both herbaceous plants and woody species. Tiller bud development is also regulated by natural factors such as temperature, planting density, moisture, and fertilizer supply [9]. Plant hormones, including auxin (IAA), cytokinin (CK), gibberellic acid (GA), abscisic acid (ABA), strigolactone (SL), and jasmonic acid (JA), play central roles in regulating the plant branching capacity. These phytohormones directly or indirectly influence branching through their biosynthesis, transport, and signal transduction pathways [10,11]. IAA is primarily synthesized in shoot tips and young leaves and transported basipetally. It suppresses lateral bud growth via apical dominance; when the shoot apex is removed, this inhibitory effect is relieved, and lateral buds can develop into new shoots [12]. CK is mainly synthesized in the roots and transported upward through the xylem via transpiration. As key regulators of lateral bud growth, CK can directly enter lateral buds to promote their outgrowth [13] and alleviate IAA-mediated apical dominance. By stimulating the germination and elongation of lateral buds, CK influences branch growth, development, and number [14,15]. In addition, they indirectly support sustained branch growth by delaying stem senescence and maintaining cell viability [16]. During lateral bud development, IAA and CK exhibit strong antagonism, and IAA further influences branching by regulating CK biosynthesis [17,18]. GA generally inhibits branching, particularly axillary bud development, and this effect is closely related to endogenous GA levels [19,20]. In apple, the GA content is positively correlated with internode length, and GA levels in the shoot tips and leaves of spur-type mutants are significantly lower than those in non-spur-type varieties. Exogenous GA application promotes shoot growth and internode elongation, whereas GA biosynthesis inhibitors suppress internode growth [21]. SL and ABA also inhibit tiller bud growth and development [22,23]. Numerous studies have shown that tillering and branching are not controlled by a single hormone but instead emerge from the balanced interaction of multiple hormones [24]. Because many hormones and interconnected pathways are involved, the regulatory network is highly complex, and no unified model has yet been established to clearly explain the relationships between different hormones and tillering or branching [25]. The formation of tillers and branches is coordinately controlled by multiple genes, and tiller bud growth is closely related to fine-scale molecular regulation [26], further highlighting the complexity of the branching regulatory network.

Metabolic processes also play crucial roles in plant growth and development, and carbon, nitrogen, and phosphorus metabolism are particularly important [27,28,29]. In carbohydrate metabolism, glucose and sucrose not only serve as energy sources but also act as signaling molecules that regulate cellular activities at multiple levels, from transcription to translation, and participate in plant morphogenesis [30]. Nitrogen, an essential macronutrient, directly affects photosynthesis and branching growth through its abundance and chemical properties [31]. It also forms a complex regulatory network with plant hormones to control development [32]. For example, under low-nitrogen conditions, the expression of the cytokinin oxidase gene *CKX* is upregulated, leading to reduced endogenous CK levels, which in turn promote root growth and branching in *Arabidopsis thaliana* [33]. Nitrogen is thus critical for lateral branch development, partly by influencing the synthesis and transport of IAA, CK, and SL. Nitrogen deficiency can enhance IAA signaling and increase SL biosynthesis, ultimately reducing the number of lateral branches [15]. Phosphorus is another key element in plant development, and phosphorus deficiency slows growth and reduces the number of branches [34]. Moreover, many studies have indicated that, in addition to nitrogen and phosphorus, nutrient elements such as potassium, boron, zinc, and arsenic also influence branching growth [35]. The metabolic pathways associated with these nutrient elements interact with one another and cooperate with hormone regulatory networks to jointly shape plant growth and morphogenesis.

The spur-type trait is an important agronomic characteristic for improving tree architecture in fruit crops. Spur-type mutations have been identified in several fruit trees, including apple, pear, peach, and citrus. Research on spur-type traits in apple is among the most systematic, and relevant findings have been widely applied in production [36,37,38,39]. Spur-type apple exhibits a strong central leader, with short shoots densely clustered along the main branches and fruit mainly borne on these short branches. Their internodes are distinctly shortened and thickened, and the trees are relatively dwarfed. These varieties show early fruiting, produce high yields, and are easy to manage [40,41]. Such traits are well suited to dwarfing and high-density planting systems, enabling early cropping and high yields while greatly enhancing planting density and management efficiency [42]. Consequently, spur-type varieties have high application value in the apple industry. Most spur-type apples have been obtained through bud sport selection, with a smaller proportion derived from mutagenesis and hybridization. Multiple studies have investigated the formation of the spur-type trait in apple from physiological, genetic, transcriptomic, and epigenetic perspectives [43]. Bud sport formation in spur-type apple is associated with gene mutations, DNA methylation changes, and retrotransposon transposition [44,45]. In spur-type varieties, deletion of a 167 bp sequence in the promoter region of the *MdTCP11* gene, together with altered methylation, affects *MdTCP11* expression. *MdTCP11* directly binds to the promoter of *MdGA2ox8* and activates its expression, promoting the degradation of bioactive GA, inhibiting internodal cell elongation, and thereby generating the spur-type phenotype [46]. In addition, clonal variation in apple has been linked to transposon insertions and recombination between retrotransposons, and the retrotransposon *atrl* underlies variation in ‘Delicious’ spur-type cultivars [47]. Moreover, *MdWUS2* has been shown to regulate branching by repressing the expression of *MdTCP12* [48].

Despite significant progress in understanding plant branching regulation, the molecular genetic basis underlying spur-type branching in apple remains poorly characterized. Existing studies on apple single nucleotide polymorphism (SNP)-trait associations have primarily focused on fruit quality or disease resistance, with limited systematic investigations into branching habit, leaving a gap in linking genomic variations to the spur-type development. Based on this research gap, we propose a testable hypothesis: Spur-type and non-spur-type apple exhibit distinct SNP patterns at the whole-genome level, and the candidate genes harboring these SNPs may be associated with spur-type traits in apple. To address these challenges, we constructed a hybrid segregation population using two high-quality spur-type cultivars, ‘Miyazaki Spur Fuji’ and ‘Starkrimson’, as parents. After years of phenotypic selection, we obtained progeny with a relatively uniform genetic background but stable phenotypic differences in spur versus non-spur traits. Plants of the typical spur-type line ‘0301-13-14’ and the non-spur-type line ‘0301-50-32’ were ultimately selected as experimental materials. Through phenotypic trait evaluation and anatomical observation, key phenotypic characteristics including shoot length, internode length, stem diameter, bud morphology, and shoot anatomical structure of the two lines were compared, clarifying the phenotypic differences and cellular features between spur-type and non-spur-type lines. Using the Illumina NovaSeq X Plus^TM^ platform (Illumina, San Diego, CA, USA), we performed whole-genome resequencing to systematically identify genomic variant sites. Specific research objectives include: identifying genome-wide single nucleotide polymorphisms (SNPs) and insertions/deletions (InDels) in representative spur-type and non-spur-type apple lines with the same genetic background; screening candidate genes associated with branching characteristics through functional annotation of variant genes and Kyoto Encyclopedia of Genes and Genomes (KEGG) pathway enrichment analysis; and ultimately establishing associations between key genetic variations, enriched pathways, and anatomical differences in branching traits. The findings of this study will provide a direct theoretical basis for the identification of functional genes related to the spur-type trait and further clarify the developmental regulatory mechanisms underlying the spur-type trait in apple on a genome-wide scale. They also have implications for overcoming bottlenecks in the breeding of spur-type varieties and for promoting the implementation of molecular breeding technologies for apple improvement.

## 2. Materials and Methods

### 2.1. Plant Materials

In 2003, the Apple Research Group of the Changli Institute of Pomology, Hebei Academy of Agriculture and Forestry Sciences, Changli, China performed a sexual hybridization using ‘Miyazaki Spur Fuji’ and ‘Starkrimson’ as parents. After years of systematic selection and evaluation, the non-spur-type line ‘0301-50-32’ (designated as NSP) and the spur-type line ‘0301-13-14’ (designated as SP) were identified in 2016. These elite lines were planted at the apple orchard in Changli (39°42′–39°45′ N, 119°10′–119°13′ E), China. The orchard at this site has relatively deep soil, excellent nutrient and water conditions, and trees in a state of vigorous growth.

**Figure 1:**
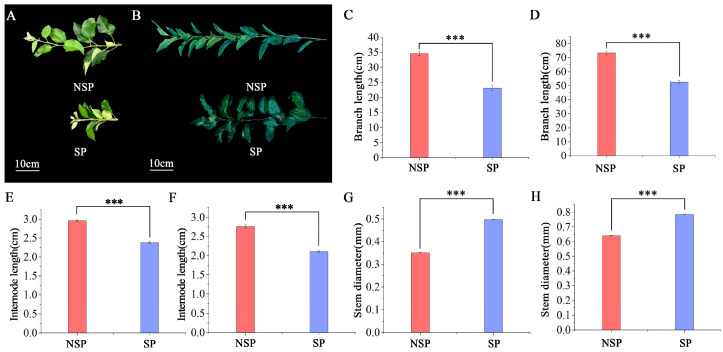
Growth differences in current-year shoots between non-spur-type (NSP) and spur-type (SP) during the spring rapid-growth period and the summer growth-cessation period. New-shoot branching phenotypes of NSP and SP during the spring rapid-growth period (**A**) and the summer growth-cessation period (**B**). Branch length (**C**), internode length (**E**), and stem diameter (**G**) during the spring rapid-growth period, and branch length (**D**), internode length (**F**), and stem diameter (**H**) during the summer growth-cessation period. Data are presented as mean ± standard error (SE, *n* = 3). *** indicate significance at *p* < 0.001, respectively.

In 2025, experimental materials were collected during the spring rapid-growth period (May 15; Figure 1A) and the summer growth-cessation period (July 10; Figure 1B). The materials included the NSP and the SP, both with stably expressed phenotypic traits. For convenience, these two lines are referred to as NSP and SP in the following experiments. A total of 20 plants (including 10 biological replicates for NSP and 10 for SP) were used in this study.

### 2.2. Morphometric Measurements

During the spring rapid-growth period (May 15) and the summer growth-cessation period (July 10) of the current-year shoots, three plants with uniform vigor were selected from each group (NSP and SP). From each plant, three main branches were randomly chosen, and the length, internode length, and thickness of the current-year shoots were measured. Each measurement was performed with three independent biological replicates and 2 technical replicates.

### 2.3. Anatomical and Histological Observation

During the rapid growth period in spring (May 15), one plant with uniform growth vigor was selected from each group (NSP and SP). From each selected plant, 3 samples of annual shoot stems and 3 apical bud samples were collected for NSP and SP, respectively. During the growth-cessation period in summer (July 10), 3 terminal buds were collected from both NSP and SP, respectively. Immediately after sampling, tissues were fixed in FAA solution (38% formaldehyde/glacial acetic acid/70% ethanol = 1:1:18) (Servicebio, Wuhan, China) at 4 °C for 7 days.

Following fixation, samples were dehydrated through an ethanol series of 75%, 85%, 90%, 95%, and twice in 100% ethanol (China Pharmaceutical Group Chemical Reagents, Beijing, China) (30 min for each step). They were then infiltrated with 100% ethanol, a 1:1 mixture of ethanol and xylene (China Pharmaceutical Group Chemical Reagents, Beijing, China), and pure xylene (30 min for each step), and embedded in paraffin. Sections (8–10 µm thick) were prepared using a Leica RM 2016 microtome (Shanghai Leica Instruments, Shanghai, China). After dewaxing in Dewaxing Solution I/II (Servicebio, Wuhan, China) (20 min each at 37 °C) and rehydration through a graded ethanol series, sections were stained with safranin (Servicebio, Wuhan, China) for 2 min at room temperature. They were then differentiated in graded ethanol, counterstained with plant solid green solution (Servicebio, Wuhan, China) for 6–20 s, dehydrated in absolute ethanol, cleared in xylene, and mounted with neutral gum (China Pharmaceutical Group Chemical Reagents, Beijing, China).

The tissue sections were observed under a Nikon Eclipse E100 microscope (Nikon Corporation, Tokyo, Japan) equipped with a DS-U3 imaging system. The thicknesses of the periderm, cortex, phloem, xylem, and pith, as well as cell number and cell diameter, were measured using the microscope scale and quantified from image fields with CaseViewer (v2.4) software. Two technical replicates were performed for each sample.

### 2.4. Resequencing Variant Analysis

In May 2025, three NSP plants and three SP plants with robust, uniform growth vigor were selected. From each plant, three tender young leaves from spring shoots were collected. The leaves were flash-frozen in liquid nitrogen and stored at −80 °C until use. The leaves from each individual plant were pooled prior to the separate extraction of genomic DNA from NSP and SP. Genome resequencing was performed by Majorbio (Shanghai, China) on the Illumina NovaSeq X Plus^TM^ platform using an Illumina PE150 strategy (total read length 300 bp). After raw data were generated, quality control was conducted to remove low-quality reads. Low-quality data were filtered out according to the criteria of Q30 base percentage > 80% and GC content ≥ 35%, to obtain high-quality clean data. Raw reads of low quality (mean phred score < 20), including reads containing adapter contamination and unrecognizable nucleotide (N base > 10) were trimming or discard by software Fastp (v1.0.1). Reads after trimming were mapping to their reference using BWA-MEME (v1.0.6) software under default mapping parameters. Clean reads were first aligned to the apple reference genome GDDH13_v1.1 using BWA-MEME with the MEM algorithm as the alignment method [49], for the purpose of determining their genomic localization and generating corresponding BAM alignment files. After the filtered clean reads were aligned to the reference genome, Samtools (v1.17) and Bedtools (v2.30.0) were employed to calculate genome-wide base coverage metrics. Specifically, genome coverage was defined as the percentage of the total reference genome length covered by reads, while coverage depth referred to the number of reads covering each individual base. These metrics were primarily used to evaluate the uniformity of sequencing data and their homology with the reference sequence. VCFtools (v0.1.16) was then utilized to estimate the missing site rate at both the sample and population levels. Samples with a missing rate > 15% and sites with a population-level missing rate > 10% were excluded to ensure high-quality data for subsequent analyses. Subsequently, the alignment results (BAM files) were processed following the GATK(v4.6.2.0) Best Practices pipeline, and SNP calling was performed using the GATK Haplotype method with default parameters. To control for population structure, principal component analysis (PCA) was conducted based on the high-quality SNP dataset after quality control, using GCTA with its default parameters. This step was intended to minimize the confounding effects of population stratification and genetic relatedness on the results of association analysis.

Since the branch length of apple is a quantitative trait, R software (v4.3.1) was used for inter-group comparisons. The statistical significance threshold was set at *p* < 0.05, with multiple test correction performed via the Bonferroni method. The results were expressed as “Mean ± standard deviation (Mean ± SD)”. Finally, GEMMA software (v0.98.5) was applied to perform genome-wide association analysis between SNPs and branch-related phenotypic traits. The significance threshold for associations was determined using the Bonferroni correction, and the false discovery rate (FDR) < 0.05 was additionally used as the criterion for screening candidate associated loci. Subsequently, the GATK Best Practices workflow [50] was applied to recalibrate the BAM files and detect SNPs and InDels, with variant calling performed using the GATK HaplotypeCaller algorithm. Functional annotation of SNPs and InDels was conducted with SnpEff (v4.3) [51], including annotation of genomic locations and predicted functional impacts. Based on their effects on protein-coding sequences, variants were classified into four categories in descending order of severity: high, moderate, low, and modifier. High denotes variants with severe functional impacts, moderate indicates variants with moderate effects, low refers to variants with minor effects, and modifier corresponds to variants with no predicted functional consequence. Therefore, only high- and moderate-impact variants were retained for subsequent analyses.

After identifying SNPs and InDels shared at the same genomic positions in SP and NSP, genes harboring deleterious mutations, such as nonsynonymous mutations, frameshift mutations, and stop-codon losses that affect gene function, were retrieved from the differential variant set. DIAMOND was then used to align these genes against the NR, UniProt, and Gene Ontology databases for functional prediction. In addition, KOBAS was used to align these genes against the KEGG database and annotate associated metabolic pathways (Figure S1).

### 2.5. Statistical Analysis

The results are presented as means ± SE of three replicates. Statistical analyses were performed using IBM SPSS Statistics 23.0 (https://www.ibm.com/docs/en/spss-statistics/23.0.0, accessed on 15 June 2024). Data were analyzed using Student’s *t*-test and ANOVA, and differences were considered statistically significant at *p* < 0.05.

## 3. Results

### 3.1. Morphometric Measurements of NSP and SP

Highly significant differences in shoot phenotypic traits were observed between NSP and SP during both the spring rapid-growth period and the summer growth-cessation period. During spring, the average branch length was 34.60 cm in NSP and 23.20 cm in SP (Figure 1C). By summer, branch length had increased to 73.50 cm in NSP and 52.67 cm in SP (Figure 1D). In spring, the internode length of NSP and SP averaged 2.96 cm and 2.38 cm, respectively (Figure 1E), whereas in summer these values slightly decreased to 2.76 cm and 2.11 cm, respectively (Figure 1F). Basal stem diameter increased steadily from spring to summer. During the spring rapid-growth period, basal diameter was 0.352 cm in NSP and 0.498 cm in SP (Figure 1G), and by summer it had increased to 0.641 cm in NSP and 0.785 cm in SP (Figure 1H).

Overall, across both sampling periods, the average branch length and internode length of SP were significantly shorter than those of NSP (*p* < 0.01), whereas the average basal stem diameter of SP was significantly larger than that of NSP (*p* < 0.01). These stable phenotypic differences clearly reflect the typical characteristics of spur-type varieties (short branches, short internodes, and thick stems) and are consistent with the phenotypic index data described above.

### 3.2. Anatomical Observation and Analysis

**Figure 2:**
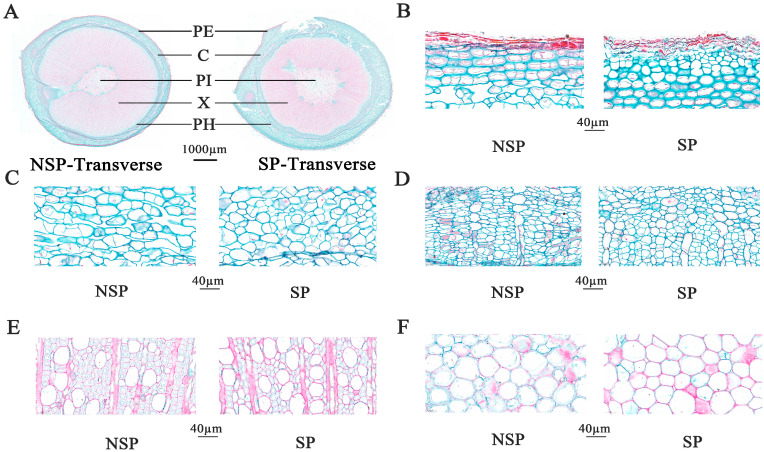
Transverse anatomical structure at the base of annual new shoots of NSP and SP during the spring rapid-growth period. Transverse sections of annual new shoots in NSP and SP (**A**). Cytological features of NSP and SP in different tissues: PE, periderm (**B**); C, cortex (**C**); PH, phloem (**D**); X, xylem (**E**); PI, pith (**F**).

To investigate the role of cell differentiation in the formation of the spur-type trait in apple, we performed systematic histological observations on transverse paraffin sections at the base of current-year shoots using the CaseViewer (v2.4) image analysis system combined with an optical microscope (Nikon Corporation, Tokyo, Japan). As shown in Figure 2A, periderm thickness did not differ significantly between NSP and SP (*p* > 0.05). However, the thicknesses of the cortex, phloem, and pith in SP were significantly greater than those in NSP, with highly significant differences observed in cortex and phloem thickness (*p* < 0.01) and a significant difference in pith thickness (*p* < 0.05). In contrast, NSP exhibited a highly significant increase in xylem thickness compared with SP. Microscopic observation of cell morphology at 60× magnification (Figure 2B–F) revealed pronounced histological differences in cell shape and arrangement across all tissues between NSP and SP. Cells in all tissues (periderm, cortex, phloem, xylem, pith) of NSP were generally larger in size, loosely arranged with distinct intercellular gaps. In particular, cortical and phloem cells of NSP displayed an irregular laterally elongated morphology, and their cell diameters were significantly larger than those of SP. Conversely, cells in all tissues of SP were smaller, predominantly round or elliptical in shape, with narrow intercellular gaps and a more compact arrangement. These results confirm that NSP and SP differ substantially in tissue composition ratio and cell morphological characteristics in the transverse sections of shoot bases, and these histological variations may serve as the key cytological basis for regulating branching divergence in apple.

**Figure 3:**
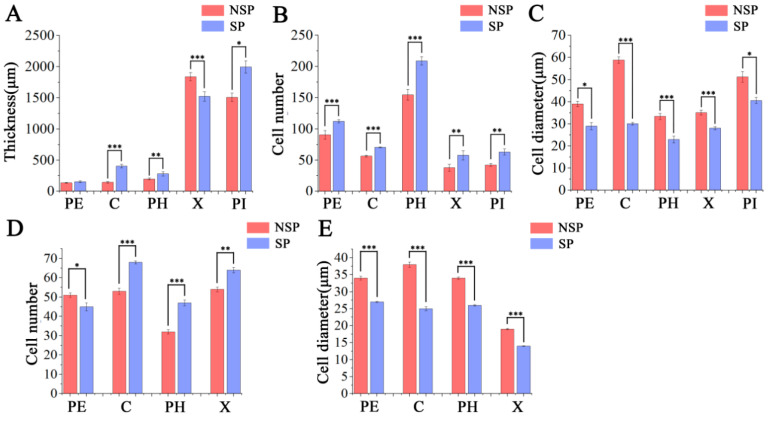
Statistical analysis of cytological characteristics at the base of current-year shoots in NSP and SP. Tissue thickness (**A**), cell number per unit area (**B**), and cell diameter per unit area in cross-sections (**C**). Cell number per unit area (**D**) and cell diameter per unit area (**E**) in longitudinal sections. PE, periderm; C, cortex; PH, phloem; X, xylem; PI, pith. Mean values ± SE (*n* = 3). *, **, and *** indicate significance at *p* < 0.05, *p* < 0.01, and *p* < 0.001, respectively.

Quantitative analysis of the thickness of each tissue in the sections further verified the aforementioned histological differences (Figure 3A–C). The results showed that the thicknesses of the periderm, cortex, phloem, and pith in NSP were significantly lower than those in SP, whereas the xylem thickness in NSP was highly significantly greater than that in SP (*p* < 0.01). Statistical analysis of cell number revealed that, compared with SP, NSP exhibited lower cell density in all tissues (periderm, cortex, phloem, xylem, pith), with a significant reduction in the number of cells per unit area. The results of cell diameter measurement indicated that the average diameter of cells in all tissues of NSP was significantly larger than that in SP, and the differences in cell diameter of the cortex, phloem, and xylem reached a highly significant level (*p* < 0.01).

**Figure 4:**
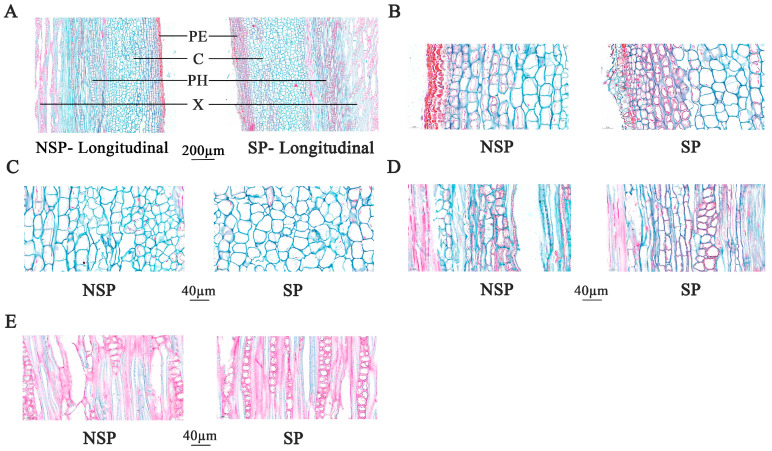
Longitudinal anatomical observation of the base of annual new shoots in NSP and SP during the spring rapid-growth period. Longitudinal sections of current-year shoots in NSP and SP (**A**). Cytological features of NSP and SP: PE, periderm (**B**); C, cortex (**C**); PH, phloem (**D**); X, xylem (**E**).

To further characterize the histological differences, we additionally performed histological analysis on longitudinal paraffin sections at the base of NSP and SP shoots (Figure 4A). Microscopic observations at 60× magnification revealed that the periderm (Figure 4B), cortex (Figure 4C), phloem (Figure 4D), and xylem (Figure 4E) of NSP all exhibited larger intercellular spaces with loosely arranged cells. By contrast, the corresponding tissues of SP had cells tightly arranged in the longitudinal direction with narrow intercellular gaps. Quantitative analysis results (Figure 3D) showed that the cell number in the periderm of NSP was significantly higher than that of SP (*p* < 0.05), whereas the cell numbers in the cortex, phloem, and xylem of NSP were highly significantly lower than those of SP (*p* < 0.01). Cell diameter measurement data (Figure 3E) indicated that the cell diameters of the periderm, cortex, phloem, and xylem in NSP were all highly significantly larger than those in SP (*p* < 0.01). Collectively, the histological characteristics of transverse and longitudinal sections demonstrate that the distinct differences in cell number (density) and cell morphology (size and arrangement pattern) between NSP and SP directly contribute to the divergence in their shoot growth dynamics.

**Figure 5:**
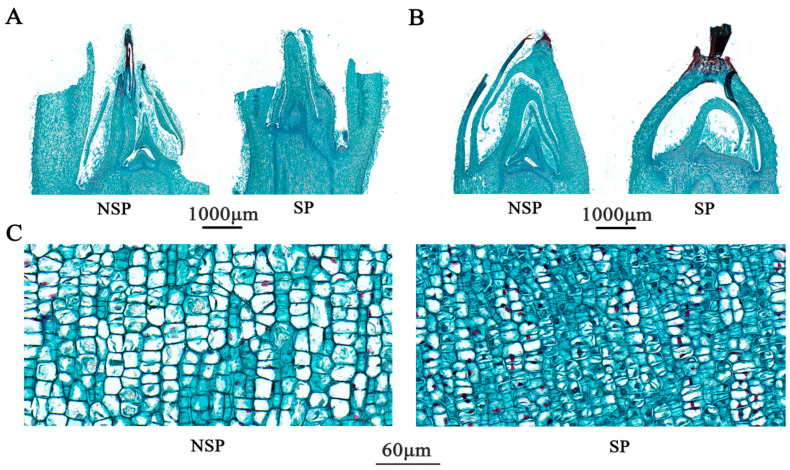
Longitudinal sections of terminal buds of NSP and SP at different developmental stages. Terminal buds during the spring rapid-growth period (**A**), terminal buds during the summer growth-cessation period (**B**), and cells of the shoot axis (**C**).

Furthermore, dynamic histological observations were conducted on longitudinal paraffin sections of terminal buds from annual new shoots of NSP and SP across different growth stages. During the spring rapid growth stage, histological characterization revealed (Figure 5A) that the vegetative cone of NSP terminal buds was larger in volume and plump in morphology, with more young leaves and leaf primordia differentiated around it. Some leaf primordia had gradually differentiated into scale leaves. In contrast, the vegetative cone of SP terminal buds was smaller, with a greater number of bud primordia than NSP, and the differentiation process of leaf primordia was relatively slow. Observations of bud axis cell morphology at 50× magnification (Figure 5C) indicated that the longitudinal diameter of bud axis cells in NSP was significantly larger than that in SP, whereas SP bud axis cells were characterized by smaller diameter and greater cell number. After entering the summer growth cessation stage, NSP and SP terminal buds exhibited distinctly different histological states (Figure 5B). The NSP terminal buds were shrunken and slender, with incompletely developed and loosely arranged outer scale leaves. Notably, cells in the (shoot apical meristem) (SAM) region maintained a certain level of mitotic activity, allowing vegetative growth to proceed continuously. By contrast, the SP terminal buds were plump and rounded, covered by multiple layers of tightly arranged outer scale leaves, cell division in the SAM almost completely ceased, and the terminal buds entered dormancy prematurely.

### 3.3. Variant Analysis of Resequencing Data in the Hybrid Progeny of NSP and SP

The raw data for this study were obtained from the initial sequencing output of the Illumina NovaSeq X Plus^TM^ platform and consisted of short sequence reads and total base counts for each sample. Sequencing data from the two apple samples are summarized in Table 1. For NSP, 7.42 Gb of clean bases and 24,635,645 clean reads were generated, with 91.10% of bases at or above Q30 and a GC content of 38.68%. For SP, 8.21 Gb of clean bases and 27,259,601 clean reads were obtained, with 92.39% of bases at or above Q30 and a GC content of 38.79%.

Quality control analysis showed that, aside from a normal imbalance in the first few bases of the reads, there was essentially no separation between A/T and G/C in base composition, and the sequencing base error rate remained below 0.001%. The apple reference genome used has a chromosome-level assembly with a total size of 676.69 Mb. Alignment of the sequencing reads to this reference yielded an average mapping rate of 97.82% for NSP and SP. The average sequencing depth of the two samples across the reference genome (excluding N regions) was approximately 10×, with individual depths of 12.23× for NSP and 13.62× for SP. These results indicate that the coverage of the reference genome was thorough, with a high number of detectable variant sites, even coverage depth, and highly random sequencing across the genome.

### 3.4. Detection and Annotation of SNPs

**Figure 6:**
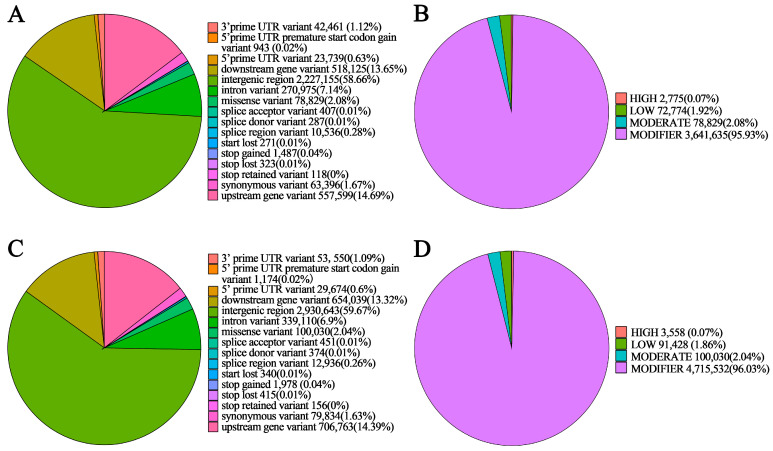
Annotation of the detected single-nucleotide polymorphisms (SNPs) in NSP and SP. SNP functional distribution in NSP (**A**) and SP (**C**); distribution of SNP functional effects in NSP (**B**) and SP (**D**).

Analysis of mutation-detection results showed that a total of 2,712,688 SNPs were identified in NSP, including 1,858,683 transitions and 858,033 transversions, with a transition/transversion (Ti/Tv) ratio of 2.17; 77.32% of these SNPs were heterozygous. In SP, 3,538,106 SNPs were detected, comprising 2,434,493 transitions and 1,110,116 transversions, with a Ti/Tv ratio of 2.19; 60.38% of these SNPs were heterozygous (Table 2). In NSP, 2775 and 78,829 SNPs were predicted to have severe and moderate effects on protein function, respectively (Figure 6B). In SP, 3558 and 100,030 SNPs were predicted to have severe and moderate effects on protein function, respectively (Figure 6D). The Ti/Tv ratios of SP and NSP (2.17/2.19) were both within the normal range of the apple genome, indicating that the SNP calling results had no significant false-positive bias, and the underlying data were reliable and suitable for subsequent analyses.

Most variants in both NSP and SP were located in intergenic regions, accounting for 58.66% and 59.67% of the total, respectively (Figure 6A,C). Within coding sequence (CDS) regions, NSP contained 80,910 nonsynonymous mutations (amino acid changes in the coding region) and 63,396 synonymous mutations (no amino acid change), whereas SP harbored 102,763 nonsynonymous mutations and 79,834 synonymous mutations (Table 3). Although SNPs in intergenic regions accounted for a higher proportion, SNP variations in coding sequence (CDS) regions, especially nonsynonymous mutations, directly alter protein sequences and exert a more direct effect on phenotypes. The number of SNPs in the CDS regions of SP (182,597) was higher than that of NSP (144,306), with a relatively higher proportion of nonsynonymous mutations (SP: 102,763/182,597 ≈ 56.3%; NSP: 80,910/144,306 ≈ 56.1%). These results indicated that the protein-coding regions of SP were subjected to stronger selection pressure, which is consistent with the characteristics of directional selection for the spur phenotype.

### 3.5. Detection and Annotation of InDels

**Figure 7:**
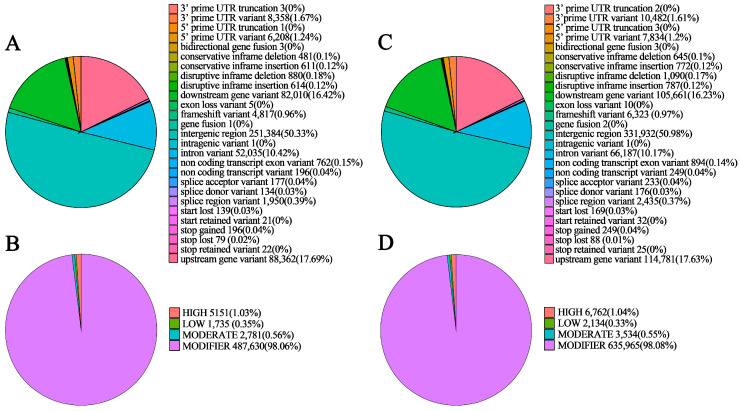
Annotation of detected InDels in NSP and SP. InDel functional distribution in NSP (**A**) and SP (**C**), and distribution of InDel functional effects in NSP (**B**) and SP (**D**).

NSP contained a total of 319,195 InDels, including 148,304 insertions and 170,891 deletions, with a heterozygous ratio of 75.55%. In SP, 415,521 InDel sites were detected, comprising 194,940 insertions and 220,581 deletions, with a heterozygous mutation ratio of 60.0% (Table 4). Among these, NSP harbored 5151 InDel sites predicted to severely affect protein function and 2781 InDel sites with moderate functional impacts (Figure 7B); SP contained 6762 InDel sites with severe effects on protein function and 3534 InDel sites with moderate functional impacts (Figure 7D).

Most variants in both NSP and SP were located in intergenic regions, accounting for 50.33% in NSP (Figure 7A) and 50.98% in SP (Figure 7C). However, InDels with strong predicted effects on gene function were mainly concentrated in CDS regions. Further analysis of differential variation patterns revealed that CDS-associated InDels in NSP and SP not only displayed substantial diversity in variant types but also differed markedly in number. Specifically, SP contained 6323 frameshift variants, significantly more than the 4817 frameshift variants detected in NSP, and a similar quantitative trend was observed for stop-gained variants. Notably, SP had more high-impact variants, including disruptive in-frame InDels and frameshift mutations, than NSP (Table 5). When such high-impact variants occur in the CDS of branching-related genes, they are highly likely to modulate spur-type traits by disrupting normal protein function. The total number of InDels in SP (415,521) was statistically significantly higher than that in NSP (319,195), while its heterozygosity rate (60.0%) was significantly lower than that of NSP (75.55%). This indicated that as a non-selected germplasm, NSP retains greater wild-type genetic diversity, and its InDels mostly consist of neutral or weakly functional variations that exert no critical effects on the branching phenotype.

### 3.6. Gene Ontology (GO) Annotation

**Figure 8:**
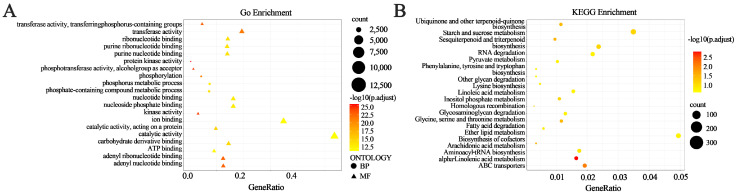
Analysis of differential gene expression between NSP and SP. The variant genes enriched in the top 20 Gene Ontology terms (**A**), categorized into molecular function (MF) and biological process (BP). The variant genes enriched in the top 20 KEGG pathways (**B**).

Based on the differential variation analysis of NSP and SP, a total of 29,157 genes carrying high- and moderate-impact deleterious mutations were identified. To further characterize the functions of these variant genes, we performed GO term mapping and enrichment analysis. The results revealed significant enrichment for 20 GO terms (Figure 8A), which were mainly classified into two major categories. In the Molecular Function (MF) category, variant genes were primarily associated with transferase activity and catalytic activity, whereas in the Biological Process (BP) category, they were mainly involved in phosphorylation and phosphorus metabolic processes.

### 3.7. Pathway Enrichment Analysis

To further investigate the functions of the mutant genes, variant genes were annotated and subjected to KEGG enrichment analysis. A total of 29,157 variant-related genes were selected as the input dataset, which were derived from the annotation results of SNPs and InDels identified in NSP and SP (including genes with functional SNPs/InDels, such as nonsynonymous mutations, frameshift variants, and promoter variants. These genes were mainly assigned to two categories, environmental information processing and metabolism, and “metabolic pathways” were the most highly represented (Figure 8B). A total of 20 pathways were enriched, among which 6 reached statistical significance (adjusted *p* < 0.05). These significantly enriched pathways included α-linolenic acid metabolism (ko00592), ABC transporters (ko02010), sesquiterpenoid and triterpenoid biosynthesis (ko00909), arachidonic acid metabolism (ko00590), fatty acid degradation (ko00071), and ubiquinone and other terpenoid-quinone biosynthesis (ko00130). Among these pathways, α-linolenic acid metabolism (ko00592) showed the most significant enrichment. Furthermore, the starch and sucrose metabolism pathway (ko00500) contained the largest number of enriched candidate genes. According to KEGG pathway annotation, 584 candidate genes were enriched in these seven pathways, corresponding to 236 distinct gene functions. Among these, 123 candidate genes were potentially associated with branching (Table S1).

## 4. Discussion

### 4.1. The Anatomical Structure of Apple Branches Is Associated with Shoot Development

In this study, SP and NSP exhibited distinct morphological differences. SP displayed phenotypic characteristics of short branches, short internodes, and thick stems, whereas NSP showed contrasting features with long branches, long internodes, and slender stems. Previous studies have indicated that shoot length in apple primarily depends on the internode length within the extension growth unit and the number of newly formed nodes [52]. Therefore, the aforementioned morphological differences directly reflect variations in the structural organization of their growth units. From an anatomical perspective, the thicknesses of the periderm, cortex, phloem, and pith in SP were all greater than those in NSP, and these tissues contained a higher number of cells. However, cells in the periderm, cortex, phloem, xylem, and pith were smaller and more compactly arranged in SP than in NSP. These results suggest that cell size and cell packing density are key anatomical factors determining internode length in apple. Furthermore, differences were observed in the activity and structure of the SAM between SP and NSP. The longitudinal diameter of bud-axis cells was significantly greater in NSP than in SP, whereas the rate of bud development was faster in SP. While cell division in the SAM of SP had nearly ceased, the SAM of NSP maintained a certain level of mitotic activity, resulting in more vigorous vegetative growth. Therefore, differences in SAM activity are also important factors influencing branches growth.

Correlations between plant architecture and cell size and number have also been identified in other species. For instance, height variation in *Oryza sativa* is closely related to cellular processes such as morphogenesis and cell division [53]. A dwarf mutant of *Brassica oleracea* exhibits enlarged cells but a reduced number of cell layers [54]. In contrast, the dwarf mutant of *Sophora davidii* shares similarities with SP in this study, characterized by small, compactly arranged cells, which contrasts with the larger, loosely arranged cells of its wild type [55]. These cross-species comparisons further support the regulatory role of cell size and tissue organization in plant branching growth.

### 4.2. Plant Hormones Are the Main Factors Affecting Branching

Plant hormones are the main factors influencing branching. The processes of their synthesis, transportation and signal transduction play a crucial role in regulating the branching ability of plants [56]. In this study, to elucidate the association between genetic pathways and apple branching development, we performed systematic functional annotation and enrichment analysis on the variant genes. KEGG pathway enrichment analysis indicated that the alpha-linolenic acid metabolic pathway was the most significantly enriched. Within this pathway, we identified a total of 28 genes associated with JA biosynthesis, including *LOX2*, *LOX2.1*, *LOX3*, *LOX6*, *OPR2*, *AOC*, and *AIM1*. α-linolenic acid is a well-known precursor for JA biosynthesis [57], which exerts a distinct negative regulatory effect on branch growth in woody plants. For instance, in *Pyrus communis* spur-type varieties exhibit significantly elevated JA levels in their shoot apices, accompanied by phenotypes of fewer branches and shorter current-year shoots [58]. We therefore speculate that deleterious SNP or InDel mutations in genes of the alpha-linolenic acid metabolism pathway may impair gene function, thereby affecting the synthesis and transport of critical hormones such as JA.

Furthermore, we found that the ATP-binding cassette (ABC) transporter pathway was also significantly enriched. As core carriers for the transmembrane transport of plant hormones, members of the ABC transporter family participate in the regulation of plant branching development by modulating the transport of phytohormones such as IAA, CK, ABA, and SL [59]. Within this pathway, we identified a total of 47 candidate genes associated with plant hormone transport. Among these, genes related to IAA transport, including *ABCB19*, *ABCB11*, *ABCB14*, *ABCB15*, and *ABCG36*, harbored high- and moderate-impact SNP or InDel mutations in both SP and NSP. The polar transport and local accumulation of IAA are crucial for regulating lateral bud germination and apical dominance [60]. Previous studies have confirmed that mutations in *ABCB19* lead to reduced IAA transport efficiency, resulting in a phenotype with increased branching in *A. thaliana* [61]. Functional abnormalities in *ABCB11*, *ABCB14*, and *ABCB15* affect vascular tissue development and IAA polar transport in the stems of *A. thaliana* [62]. Tobacco lines overexpressing *ABCG36* exhibit accelerated stem elongation and growth than the wild type, suggesting that *LkABCG36* may promote growth by enhancing the transport of auxins such as IBA and IAA [63]. Based on these findings, we speculate that variations in IAA transport-related genes in apple may affect IAA transport efficiency, thereby altering its distribution in key apple tissues.

We also identified deleterious mutations in the *ABCG14* gene, which is involved in cytokinin transport within the ABC transporter pathway. CK is a key hormone that maintains SAM activity and promotes cell differentiation and regeneration [64]. *ABCG14* is the major transporter that mediates the translocation of CK from roots to shoots. Previous studies have confirmed that A. thaliana *Atabcg14* mutants, exhibit phenotypes of slender and shorter stems and retarded shoot growth due to impaired cytokinin transport, and the size of xylem and phloem cells are markedly reduced [65]. We therefore speculate that deleterious mutations in *MdABCG14* may reduce CK transport efficiency in apple, leading to a decline in the cell division activity of the SAM. This could be one of the reasons why spur-type apple shoots exhibit the characteristic feature of reduced cell size.

In addition, we have also identified the gene *ABCG25* associated with abscisic acid transport and the gene *PDR1* related to strigolactone transport in the ABC transporter pathway. Both genes harbored deleterious mutations in both SP and NSP. *ABCG25* has been shown to function as a high-affinity ABA transporter, and ABA can affect branch growth by inhibiting cell elongation [66]. *PDR1* is a key transporter for SL, studies have confirmed that *Petunia hybrida pdr1* mutants exhibit significantly stronger lateral branching compared to the wild type due to impaired SL transport [67]. Additionally, ABA and SL regulate tillering at different nodes in rice and act synergistically to inhibit branching [68]. Therefore, variations in *MdABCG25* and *MdPDR1* in apple are likely to affect the transport ability of ABA and SL.

### 4.3. Sucrose Signaling and Distribution Form the Material Foundation for Plant Branch Development

Based on the KEGG pathway enrichment analysis of genes harboring deleterious mutations in SP and NSP, we found that the starch and sucrose metabolism pathway contained the largest number of enriched candidate genes. This suggests that this pathway may be one of the core pathways regulating branching development in apple. Further screening revealed that this pathway contains candidate genes related to sucrose, such as *SPS1*, *BGLU11*, *SUS5*, and *SS3*, and all of which carried deleterious mutations predicted to affect their function. Sucrose phosphate synthase gene *SPS* is a key enzyme in sucrose synthesis. Enhanced *SPS* activity has been shown to promote vegetative growth and increase the number of secondary branches in *Nicotiana tabacum* and *O. sativa* [69,70]. Deleterious mutations in the *SPS1* gene may alter the enzymatic activity of its encoded protein, thereby affecting sucrose synthesis efficiency in SP and NSP. Sucrose acts not only as a core energy source for plant growth and development but also as an important signaling molecule regulating lateral bud outgrowth and internode elongation. Therefore, functional variations in these pathway genes may indirectly influence the branching phenotypes of SP and NSP by altering sucrose accumulation and partitioning patterns.

Similarly, variations in β-glucosidase *(BGLU11*) and sucrose synthases (*SUS5*, *SS3*) warrant attention. *BGLU11* may influence shoot structural development and the levels of IAA, CK, and GA by participating in cell wall metabolism or the hydrolysis of hormone glycoside precursors [71,72]. Functional validation in related species supports a role for *BGLU11* in plant architecture regulation. For instance, mutation of *bglu11* in *Cynodon dactylon* leads to shortened internodes and a significant reduction in plant height [73]. Furthermore, *BGLU11* in *Setaria italica* has been directly identified as a core candidate gene regulating axillary branching [71]. Sucrose synthases (*SUS/SS*) are involved in cellulose synthesis, carbon allocation, and cell wall formation. Enhanced *SUS* function significantly promotes xylem development and plant height growth in *Populus tomentosa* [74,75]. In summary, deleterious mutations in candidate genes of the starch and sucrose metabolism pathway may represent the molecular basis underlying the divergence in branching phenotypes between SP and NSP. These candidate genes, which harbor deleterious mutations, are promising potential targets for molecular marker-assisted breeding of spur-type apple varieties.

### 4.4. Multiple Metabolic Pathways Synergistically Modulate the Development of Plant Branching

Plant branching development is synergistically regulated by multiple metabolic pathways, which constitutes one of the core molecular mechanisms underlying plant architecture formation. Through genome-wide variation analysis of SP and NSP apples, we identified candidate genes harboring deleterious mutations in the terpenoid biosynthesis, fatty acid degradation, and ubiquinone biosynthesis pathways. These results suggest that functional variations in the aforementioned pathways may participate in the differentiation of apple branching phenotypes by perturbing their respective metabolic networks or signal transduction processes. The terpenoid biosynthesis pathway is one of the key metabolic pathways regulating plant growth and development. Its metabolites, such as phytosterols and triterpenoids, are directly involved in physiological processes including cell proliferation, signal transduction, and organ morphogenesis [76,77,78]. In this study, we identified *SQE1*, a core gene with dual functions in both phytosterol and triterpenoid biosynthesis, which harbored deleterious mutations in both SP and NSP. Previous studies have confirmed that functional abnormalities in *SQE1* lead to distinct growth defects. For instance, *A. thaliana Atsqe1* mutants exhibit phenotypes such as arrested root development and inhibited stem elongation [78]. Notably, as a crucial prerequisite for axillary bud outgrowth, stem elongation directly determines the spatial allocation for bud growth and the efficiency of nutrient acquisition. Based on this, we speculate that deleterious mutations in apple *SQE1* may indirectly inhibit axillary bud growth and elongation by blocking the normal synthesis of terpenoids, thereby participating in the regulatory divergence of branching phenotypes between SP and NSP.

As a core hub linking plant energy metabolism and hormone biosynthesis, the fatty acid degradation pathway not only supplies critical energy for plant growth and development via the β-oxidation cycle, but also serves as an important precursor pathway for JA and IAA biosynthesis [79,80]. As key regulatory hormones governing plant branching development, JA can inhibit stem elongation [58], while IAA modulates axillary bud growth by regulating polar IAA transport [81]. In this study, genome-wide analysis revealed significant enrichment of 32 candidate genes in this pathway, including *KAT*, *LACS4*, *LACS8*, and *LACS9*, with all these genes detected to carry deleterious mutations that may impair their functions. Previous studies have provided direct evidence for the functional importance of genes in this pathway, in *A. thaliana*, the *lacs4 lacs8* and *lacs4 lacs9* double mutants display reduced vigor and slender stems [82], confirming the regulatory role of *LACS* family genes in shoot growth. Based on these findings, we hypothesize that deleterious mutations in candidate genes of the fatty acid degradation pathway in apple may disrupt the β-oxidation process, indirectly impair the synthesis efficiency and signal transduction of JA and IAA, and thereby alter the transport and distribution patterns of nutrients to axillary buds. As an indispensable macronutrient for plant growth and development, potassium (K^+^) is crucial for maintaining cell turgor and osmotic homeostasis through transmembrane transport and homeostasis regulation, thereby directly influencing cell division and elongation [83]. Intracellular K^+^ uptake and transport rely on specific K^+^ channels and transporters. Among these, *KAT1*, a core member of the inward-rectifier K^+^ channel family, has been identified as a key molecule mediating K^+^ influx into plant cells [84]. In this study, deleterious mutations of the K^+^ channel key gene *KAT1* were identified in SP and NSP. The homologous gene of *KPT1* in poplar has been confirmed to mediate K^+^ entry into cells to maintain turgor pressure, directly participating in bud development [85]. We speculate that the deleterious mutations in the apple *KAT1* gene may disrupt the structural integrity or transport activity of the channel protein, impeding the efficient uptake and accumulation of K^+^ by cells, and consequently affecting cell division and elongation.

The ubiquinone biosynthesis pathway is one of the core pathways maintaining energy metabolism and signal transduction networks in plant cells. It not only plays an irreplaceable role in balancing photosynthesis and respiration [86], but also indirectly participates in plant growth and development by regulating the homeostasis of ABA and IAA [87]. As a key rate-limiting gene in this pathway, the functional integrity of *ICS* directly determines the efficiency of ubiquinone synthesis, thereby affecting the overall physiological metabolism and growth phenotypes of plants. Previous studies have confirmed that *O. sativa OsICS* knockout mutants exhibit growth-defective phenotypes such as significantly reduced plant height and leaf chlorosis [88], which clearly demonstrates the detrimental effects of *ICS* loss-of-function on plant growth.

As a core pathway governing plant cell wall construction and shoot development, lignin biosynthesis produces lignin, which is a primary structural component of cell walls that also acts as a critical determinant for sustaining shoot mechanical strength and regulating cell growth and differentiation. The metabolic homeostasis of lignin directly modulates plant branching [89]. In the present study, we further identified deleterious mutations in *4CL* and *CYP73A*, two key genes involved in lignin biosynthesis, within the ubiquinone biosynthetic pathway. Previous studies have provided direct evidence for the functional importance of these genes and their relevance to plant phenotypes: in *Populus alba* × *P. tremula var. glandulosa*, downregulated expression of the *4CL* gene results in a marked reduction in lignin content, which in turn causes shortened xylem cell length, increased cross-sectional area but decreased number of vessel cells [90]. This finding is highly consistent with the phenotypic characterization and anatomical observations of the present study: the xylem of NSP exhibits greater thickness and plumper cell morphology, with shoots significantly longer than those of SP. These results suggest that mutations in the *4CL* gene may impair lignin synthesis efficiency, thereby modulating the developmental traits of xylem cells. Furthermore, studies on the *CYP73A* (*C4H*) loss-of-function mutant in *A. thaliana* have further confirmed that the functional integrity of this gene is a prerequisite for normal lignin biosynthesis. Mutations in *AtCYP73A* lead to insufficient lignin accumulation, which disrupts the structural stability of vascular tissue cell walls and consequently impedes polar IAA transport [91]. Given that polar IAA transport is a core regulatory process governing axillary bud germination and lateral shoot elongation, this mechanism provides a theoretical basis for the potential involvement of the *CYP73A* gene in mediating branching phenotype differentiation in apple.

This study provides valuable theoretical insights and candidate gene resources for elucidating the molecular regulatory mechanisms underlying branch formation in spur-type apple. However, considering the practical design and technical approaches adopted, several limitations remain to be addressed in future research. In terms of sampling strategy, the experimental materials were collected from a single ecological region and only included a limited number of SP and NSP apple cultivars. This constraint renders it difficult to directly extend the generalizability of the study results to diverse apple germplasm resources grown under different climatic and soil conditions, and fails to fully reflect the interactive effects of ecological environments and genetic backgrounds on branch phenotypes. Furthermore, current research on SP apples has predominantly focused on bud sport mutants of the Fuji and Red Delicious series, and the present study also centered on the hybrid progeny derived from the cross between ‘Miyazaki Spur Fuji’ and ‘Starkrimson’. Owing to the relatively homogeneous genetic background of these two parents and the exclusion of wild apple germplasm as well as distant and close relatives, this study might have overlooked certain rare variations and key genes regulating branching traits under other genetic backgrounds. At the level of molecular marker analysis, the study only focused on the detection of SNPs and InDels, while neglecting the exploration of structural variations (SVs) such as copy number variations, chromosomal inversions, and translocations, which may also play potential roles in modulating branch phenotypes. Moreover, the functional impacts of the identified mutation loci were solely predicted by bioinformatics software, without validation through functional assays of genes. As a result, the causal relationship between genotype and phenotype lacks direct experimental evidence. Based on the aforementioned limitations and current demands of apple molecular breeding, future research should actively introduce wild germplasm resources to broaden the genetic background, integrate multi-omics technologies to comprehensively identify novel regulatory genes, and enrich the pool of favorable allelic variations. Meanwhile, efforts should be made to accelerate the construction and application of molecular breeding systems: specifically, develop co-dominant markers such as cleaved amplified polymorphic sequences (CAPS) and kompetitive allele-specific PCR (KASP) based on the key SNP and InDel loci within the core genes of the pathways identified in this study, thereby establishing an efficient marker-assisted selection (MAS) system. Given that the spur trait is a quantitative trait controlled by multiple genes, the selection efficiency of a single marker may be limited. Therefore, in practical breeding programs, it is recommended to perform combined screening of multiple favorable allelic variations identified in this study and pyramid beneficial genes via MAS technology, so as to cultivate new apple varieties with more significant dwarfing effects and more compact tree architectures. In addition, CRISPR/Cas9 and other gene-editing technologies can be employed to precisely modify deleterious mutation loci in key genes or regulate their expression patterns in a targeted manner, thereby achieving precise improvement of apple branch architecture and providing a more efficient technical pathway for spur-type apple breeding.

## 5. Conclusions

Our study investigated the branching traits of spur-type apple via anatomical observations and whole-genome resequencing. Anatomical analysis revealed that the branching characteristics of spur-type apple are associated with their specific anatomical structure. The shortened internodes of SP result from reduced cell size and increased packing density, whereas the vigorous vegetative growth of NSP is also correlated with high SAM division activity. In this study, we utilized whole-genome resequencing technology to systematically analyze the genetic variations between SP and NSP. Based on functional annotation and pathway enrichment analysis of variant genes, six pathways were found to be significantly enriched (*p* < 0.05): alpha-linolenic acid metabolism, ABC transporters, sesquiterpenoid and triterpenoid biosynthesis, arachidonic acid metabolism, fatty acid degradation, and ubiquinone and other terpenoid-quinone biosynthesis. Furthermore, the starch and sucrose metabolism pathway contained the largest number of enriched candidate genes. A total of 584 candidate genes were identified across these pathways, among which 123 genes potentially correlate with branching development. However, the specific functions of these genes in the formation of apple spur traits require further validation. Collectively, the results of this study provide insights into the molecular mechanisms underlying the branching characteristics of spur-type apple and will facilitate future breeding improvements. This is crucial for the advancement of high-density, dwarfing cultivation systems in the apple industry.

## Figures and Tables

**Table 1:** Analysis of sequencing data for non-spur-type (NSP) and spur-type (SP).

Sample ID	Clean Bases (bp)	Clean Reads	Clean GC (%)	Clean Q30 (%)	Mapped Ratio (%)	Real Depth ×	Coverage (%) (≥1×)	Coverage (%) (≥4×)
NSP	7,422,980,792	24,635,645	38.68	91.10	98.05	12.23	83.91	75.90
SP	8,212,655,150	27,259,601	38.79	92.39	97.58	13.62	82.94	75.71

**Table 2:** Genetic variation in single-nucleotide polymorphisms (SNPs) detected in NSP and SP.

Sample ID	SNP Number	Transition	Transversion	Ti/Tv	Homozygosity	Heterozygosity	Het- Ratio %	High	Moderate
NSP	2,712,688	1,858,683	858,033	2.17	2,291,280	2,097,484	77.32	2775	78,829
SP	3,538,106	2,434,493	1,110,116	2.19	1,465,862	2,136,474	60.38	3558	100,030

**Table 3:** Summary of the number of SNPs in different functional categories in the coding sequence (CDS) regions of NSP and SP.

Sample ID	CDS SNPs Number	Missense Variant	Synonymous Variant	Stop Gained	Stop Lost	Start Lost
NSP	144,306	78,829	63,396	1487	323	271
SP	182,597	100,030	79,834	1978	415	340

**Table 4:** Summary of insertions/deletions (InDels) detected in NSP and SP.

Sample	Total	Insertion	Deletion	Homo	Het	High	Moderate
NSP	319,195	148,304	170,891	78,031	241,164	5151	2781
SP	415,521	194,940	220,581	166,195	249,326	6762	3534

**Table 5:** Summary of numbers of different InDel mutation types in the CDS regions of NSP and SP.

Sample ID	Conservative In-Frame Deletion	Conservative In-Frame Insertion	Disruptive In-Frame Deletion	Disruptive In-Frame Insertion	Frameshift Variant	Stop Gained	Stop Lost	Start Lost
NSP	481	611	880	614	4817	196	79	139
SP	645	772	1090	787	6323	249	88	169

## Data Availability

The original contributions presented in the study are included in the article/Supplementary Materials. Further inquiries can be directed to the corresponding author.

## Supplementary Materials

The following supporting information can be downloaded at https://www.mdpi.com/article/10.3390/genes17010096/s1, Table S1: Candidate genes related to branching; Figure S1: Schematic diagram of the whole genome resequencing process.
