# Supplementary material for: Structural and Genomic Bases of Branching Traits in Spur-Type Apple: Insights from Morphology and Whole-Genome Resequencing
Source: Genes (Basel). 2026 Jan 18;17(1):96. doi: 10.3390/genes17010096 (PMC12841532; doi:10.3390/genes17010096)
Supplement: Supplementary file 1 [file genes-17-00096-s001.zip › genes-4091557-supplementary.pdf]

Table S1 Candidate genes related to branching

Pathway	Abbreviation	Gene ID	Chromosome	Gene
α-linolenic acid metabolism	LOX2	MD07G1003600	chr8	Lipoxygenase 2
	LOX2.1	MD11G1023100	chr12	linoleate 13S-lipoxygenase 2-1
	LOX2.1	MD03G1021200	chr4	linoleate 13S-lipoxygenase 2-1
	LOX2.1	MD02G1317500	chr3	Linoleate 13S-lipoxygenase 2-1
	LOX2.1	MD02G1317800	chr3	Linoleate 13S-lipoxygenase 2-1
	LOX2.1	MD07G1003700	chr8	Linoleate 13S-lipoxygenase 2-1
	LOX2.1	MD07G1003900	chr8	Linoleate 13S-lipoxygenase 2-1
	LOX2.1	MD07G1004500	chr8	Linoleate 13S-lipoxygenase 2-1
	LOX2.1	MD07G1004600	chr8	Linoleate 13S-lipoxygenase 2-1
	LOX3	MD10G1294000	chr11	Lipoxygenase 3
	LOX6	MD13G1114000	chr14	Lipoxygenase 6
	LOX6	MD16G1113200	chr17	Lipoxygenase 6
	OPR2	MD08G1216100	chr9	12-oxophytodienoate reductase 2
	OPR2	MD08G1216200	chr9	12-oxophytodienoate reductase 2
	OPR2	MD15G1400700	chr16	12-oxophytodienoate reductase 2
	OPR2	MD15G1401300	chr16	12-oxophytodienoate reductase 2
	OPR2	MD15G1402700	chr16	12-oxophytodienoate reductase 2
	OPR2	MD15G1402800	chr16	12-oxophytodienoate reductase 2
	OPR2	MD15G1403000	chr16	12-oxophytodienoate reductase 2
	AOC	MD09G1084600	chr10	Allene oxide cyclase
	AOC	MD04G1210800	chr5	Allene oxide cyclase
	AOC	MD16G1047500	chr17	Allene oxide cyclase
	AOC	MD12G1225100	chr13	Allene oxide cyclase
	AOC	MD13G1045900	chr14	Allene oxide cyclase
	AIM1	MD15G1181900	chr16	Peroxisomal fatty acid beta-oxidation multifunctional protein AIM1
	AIM1	MD02G1043100	chr3	Peroxisomal fatty acid beta-oxidation multifunctional protein AIM1
	AIM1	MD02G1043200	chr3	Peroxisomal fatty acid beta-oxidation multifunctional protein AIM1
	AIM1	MD02G1043300	chr3	peroxisomal fatty acid beta-oxidation multifunctional protein AIM1-like
ABC transporters	PDR1	MD10G1175000	chr11	Pleiotropic drug resistance protein 1
	PDR1	MD15G1117800	chr16	Pleiotropic drug resistance protein 1

	PDR1	MD15G1117900	chr16	Pleiotropic drug resistance protein 1
	PDR1	MD02G1032700	chr3	Pleiotropic drug resistance protein 1
	PDR1	MD05G1187100	chr6	Pleiotropic drug resistance protein 1
	PDR1	MD05G1187200	chr6	Pleiotropic drug resistance protein 1
	PDR1	MD05G1187400	chr6	Pleiotropic drug resistance protein 1
	PDR1	MD08G1141400	chr9	Pleiotropic drug resistance protein 1
	PDR1	MD08G1140900	chr9	Pleiotropic drug resistance protein 1
	PDR1	MD08G1141000	chr9	Pleiotropic drug resistance protein 1
	ABCB11	MD13G1138600	chr14	ABC transporter B family member 11
	ABCB11	MD13G1139100	chr14	ABC transporter B family member 11
	ABCB11	MD17G1284300	chr18	ABC transporter B family member 11
	ABCB11	MD16G1134500	chr17	ABC transporter B family member 11
	ABCB11	MD16G1134700	chr17	ABC transporter B family member 11
	ABCB11	MD16G1135100	chr17	ABC transporter B family member 11
	ABCB11	MD09G1290100	chr10	ABC transporter B family member 11
	ABCB11	MD17G1284700	chr18	ABC transporter B family member 11
	ABCB15	MD17G1042800	chr18	ABC transporter B family member 15
	ABCB15	MD03G1211200	chr4	ABC transporter B family member 15
	ABCB15	MD09G1041500	chr10	ABC transporter B family member 15
	ABCB15	MD09G1041600	chr10	ABC transporter G family member 36
	ABCB15	MD09G1041700	chr10	ABC transporter B family member 15
	ABCB15	MD09G1041900	chr10	ABC transporter B family member 15
	ABCB15	MD11G1226100	chr12	ABC transporter B family member 15
	ABCB19	MD17G1049600	chr18	ABC transporter B family member 19
	ABCB19	MD17G1149200	chr18	ABC transporter B family member 19
	ABCB19	MD09G1049400	chr10	ABC transporter B family member 19
	ABCB19	MD09G1177100	chr10	ABC transporter B family member 19
	ABCG36	MD09G1203500	chr10	ABC transporter G family member 36
	ABCG36	MD09G1204000	chr10	ABC transporter G family member 36
	ABCG36	MD09G1204200	chr10	ABC transporter G family member 36
	ABCG36	MD17G1184700	chr18	ABC transporter G family member 29-like
	ABCB14	MD15G1010400	chr16	ABC transporter B family member 14
	ABCB14	MD01G1232000	chr2	ABC transporter B family member 14
	ABCG25	MD05G1042200	chr6	ABC transporter G family member 25

	ABCG25	MD10G1048700	chr11	ABC transporter G family member 25
	ABCG14	MD15G1000600	chr16	ABC transporter G family member 14
	ABCG5	MD15G1134300	chr16	ABC transporter G family member 5
sesquiterpenoid and triterpenoid biosynthesis	SQE1	MD10G1114800	chr11	Squalene monooxygenase SE1
	SQE1	MD15G1064100	chr16	Squalene monooxygenase SE1
	SQE1	MD15G1301500	chr16	Squalene monooxygenase SE1
	SQE1	MD00G1080800	chr1	Squalene monooxygenase SE1
	SQE1	MD02G1190300	chr3	Squalene monooxygenase SE1
	SQE1	MD02G1272900	chr3	Squalene monooxygenase SE1
	SQE1	MD05G1111800	chr6	Squalene monooxygenase SE1
	SQE1	MD05G1111900	chr6	Squalene monooxygenase SE1
	SQE1	MD05G1112100	chr6	Squalene monooxygenase SE1
	SQE1	MD07G1041500	chr8	Squalene monooxygenase SE1
	SQE1	MD07G1041600	chr8	Squalene monooxygenase SE1
	SQE1	MD07G1041700	chr8	Squalene monooxygenase SE1
	SQE1	MD07G1041900	chr8	Squalene monooxygenase SE1
	SQE1	MD07G1042000	chr8	Squalene monooxygenase SE1
	SQE1	MD08G1076800	chr9	Squalene monooxygenase SE1
fatty acid degradation	LACS4	MD14G1128200	chr15	Long chain acyl-CoA synthetase 4
	LACS4	MD08G1147500	chr9	Long chain acyl-CoA synthetase 4
	LACS4	MD02G1279100	chr3	Long chain acyl-CoA synthetase 4
	LACS4	MD02G1279200	chr3	Long chain acyl-CoA synthetase 4
	LACS4	MD06G1106100	chr7	Long chain acyl-CoA synthetase 4
	LACS8	MD09G1129700	chr10	Long chain acyl-CoA synthetase 8
	LACS9	MD15G1349800	chr16	Long chain acyl-CoA synthetase 9
	KAT1	MD05G1179100	chr6	3-ketoacyl CoA thiolase 1
	KAT1	MD06G1073900	chr7	3-ketoacyl CoA thiolase 1
	KAT1	MD10G1167800	chr11	3-ketoacyl CoA thiolase 1
ubiquinone and other terpenoid-quinone biosynthesis	4CLL9	MD13G1113700	chr14	4-coumarate--CoA ligase-like 9
	4CLL9	MD14G1161200	chr15	4-coumarate--CoA ligase-like 9
	4CLL9	MD14G1161300	chr15	4-coumarate--CoA ligase-like 9
	4CLL9	MD16G1112900	chr17	4-coumarate--CoA ligase-like 9
	4CLL9	MD16G1113000	chr17	4-coumarate--CoA ligase-like 9
	4CLL9	MD06G1147300	chr7	4-coumarate--CoA ligase-like 9

	4CLL9	MD06G1147500	chr7	4-coumarate--CoA ligase-like 9
	ICS2	MD14G1195300	chr15	Isochorismate synthase 2
	ICS2	MD14G1195400	chr15	Isochorismate synthase 2
	ICS2	MD14G1195500	chr15	Isochorismate synthase 2
	ICS2	MD06G1188700	chr7	Isochorismate synthase 2
	CYP73A14	MD00G1221400	chr1	Trans-cinnamate 4-monooxygenase
	CYP73A14	MD03G1051000	chr4	Trans-cinnamate 4-monooxygenase
	CYP73A14	MD03G1051100	chr4	Trans-cinnamate 4-monooxygenase
	4CLL1	MD13G1120400	chr14	4-coumarate--CoA ligase-like 1
	4CLL1	MD16G1120400	Chr17	4-coumarate--CoA ligase-like 1
	CYP73A16	MD11G1052900	chr12	Trans-cinnamate 4-monooxygenase
	CYP73A16	MD03G1051300	chr4	Trans-cinnamate 4-monooxygenase
	CYP73A4	MD03G1051400	chr4	Trans-cinnamate 4-monooxygenase
starch and sucrose metabolism	BGLU11	MD11G1100300	chr12	Beta-glucosidase 11
	BGLU11	MD11G1150300	chr12	Beta-glucosidase 11
	BGLU11	MD11G1150500	chr12	Beta-glucosidase 11
	BGLU11	MD11G1150800	chr12	Beta-glucosidase 11
	BGLU11	MD11G1151200	chr12	Beta-glucosidase 11
	SUS5	MD09G1280200	chr10	Sucrose synthase 5
	SUS5	MD17G1287000	chr18	Sucrose synthase 5
	SS3	MD10G1321600	chr11	Soluble starch synthase 3
	SPS1	MD15G1164900	chr16	Probable sucrose-phosphate synthase 1
	SPS1	MD02G1022300	chr3	Probable sucrose-phosphate synthase 1
	SPS1	MD08G1156900	chr9	Probable sucrose-phosphate synthase 1
	SPS1	MD08G1157000	chr9	Probable sucrose-phosphate synthase 1

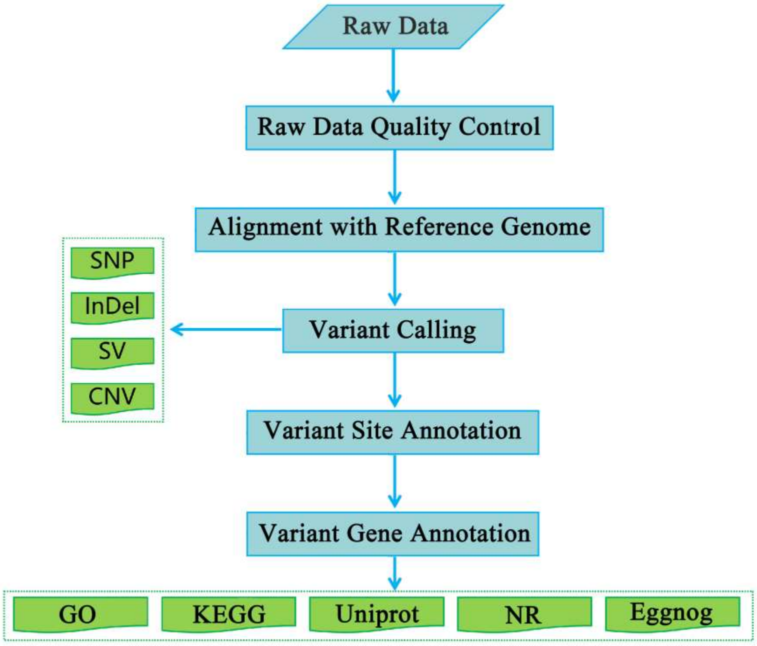

Figure S1. Schematic diagram of the whole genome resequencing process
